# The mitochondrial genome and phylogenetic position of a marine snail *Nerita* (*Heminerita*) *japonica* (Gastropoda: Neritimorpha: Neritidae)

**DOI:** 10.1080/23802359.2020.1829136

**Published:** 2020-10-21

**Authors:** Hiroaki Fukumori, Hajime Itoh, Nobuyoshi Nakajima, Yasunori Kano

**Affiliations:** a Sesoko Station, Tropical Biosphere Research Center, University of the Ryukyus, Motobu, Japan; b Atmosphere and Ocean Research Institute, The University of Tokyo, Kashiwa, Japan; c National Institute for Environmental Studies, Tsukuba, Japan

**Keywords:** Mollusca, Neritinae, Neritoidea, next-generation sequencing, phylogenetic tree

## Abstract

The mitochondrial genome of the neritid snail *Nerita* (*Heminerita*) *japonica* (Mollusca: Neritimorpha) from Kumamoto, Japan was determined by whole-genome sequencing. This mitogenome is comprised of 13 protein-coding genes, 2 ribosomal RNA (12S and 16S) genes, and 22 transfer RNA genes, with the same gene order as in the other species of the family Neritidae. A likelihood-based phylogenetic reconstruction recovered the subgenus *Heminerita* (including *N. japonica* as its type and *N. yoldii* from China) as monophyletic and sister to a clade with four species of the subgenera *Nerita* and *Theliostyla*.

The snails of the genus *Nerita* (Neritimorpha: Neritidae) are common herbivorous grazers on intertidal rocky shores in tropical, subtropical, and temperate regions worldwide. Approximately, 70 species are identified as the extant members of this genus (Frey and Vermeij 2008). A phylogenetic analysis based on partial DNA sequences of a few mitochondrial and nuclear genes has resulted in the recognition of 11 subgenera (Frey 2010), whereas relationships among the subgenera remain largely unresolved due to insufficient phylogenetic signal (Frey and Vermeij 2008).

The mitochondrial genome (mitogenome) has been assessed for its suitability for the phylogenetic reconstruction of various gastropod taxa (e.g. Uribe et al. 2016; Abalde et al. 2017; Liu et al. 2020). Six species of *Nerita* belonging to four subgenera have previously been studied in this context (Arquez et al. 2014; Feng et al. 2019; Xie et al. 2019; Castro and Colgan 2010). Here, we report the first mitogenome sequence of *Nerita* (*Heminerita*) *japonica*, the type species of *Heminerita*. This species inhabits upper intertidal rocky shores along the East Asian coast from the mainland Japan to the Korean Peninsula to Zhejiang Province, China (Frey and Vermeij 2008; Zhang et al. 2018).

A single specimen of *N.* (*H.*) *japonica* was sampled in September 2016 at Shiranui, Uki, Kumamoto, Kyushu Island, Japan (32°38′01″N, 130°38′10″E). Total DNA was extracted from its muscle tissue using a DNeasy Blood and Tissue Kit (Qiagen, Hilden, Germany) and sequenced on a Miseq System (Illumina, San Diego, CA) at the National Institute for Environmental Studies (Tsukuba, Japan). For *de novo* assembly in NOVOPlasty 3.7 (Dierckxsens et al. 2017), a partial COI fragment of the same specimen was PCR-amplified with the Folmer et al.’s (Folmer et al. 1994) primers LCO1490 and HCO2198 and Sanger-sequenced on an ABI 3130xl (Applied Biosystems, Foster City, CA) at Atmosphere and Ocean Research Institute, The University of Tokyo. A total of 3,725,719 paired-end reads from a Miseq run were assembled in NOVOPlasty with the COI sequence as a seed and with the default k-mer value of 39 (Dierckxsens et al. 2017). This resulted in a non-circular contig of 15,306 bp. The missing (tRNA^Glu^ and non-coding) region of the mitogenome was amplified with primers designed from both ends of the contig and then sequenced on ABI 3130xl. The amplified sequence had a tandem-repeat region of ca. 250 bp that prevented us to determine a complete, circularized mitogenome, although it added 571 bp to the original contig. The final mitogenome sequence was annotated using the MITOS webserver (Bernt et al. 2013) and deposited in the DNA Data Bank of Japan under the accession number LC565707. The specimen was deposited as a voucher (NJ018) in Sesoko Station, University of the Ryukyus.

This 15,877-bp mitogenome sequence contained 13 PCGs, 22 tRNAs, and two rRNAs (12S and 16S). Of the 37 genes identified, seven PCGs and eight tRNAs were encoded on the L-strand, whereas the remaining genes were encoded on the H-strand. The overall base composition was 29.8% for A, 35.4% for T, 21.2% for G, and 13.6% for C with an AT-bias. All PCGs contained ATG as the start codon, and TAA, TAG or T-- as the stop codons. NAD4 and NAD4L genes are overlapped by 7 bp. The 12S (867 bp) and 16S (1,295 bp) rRNA genes were located between tRNA^Leu^ and tRNA^Met^. The lengths of 22 tRNAs ranged from 65 to 74 bp. The gene order was the same as in the previously reported mitogenomes of neritids (Castro and Colgan 2010; Arquez et al. 2014; Fukumori et al. 2016; Uribe et al. 2016; Feng et al. 2019; Wang et al. 2019; Xie et al. 2019).

A maximum-likelihood phylogeny of the genus *Nerita* was inferred from the present and previous mitogenome data, including 13 PCG and two rRNA gene sequences from *N.* (*Heminerita*) *japonica*, *N.* (*H.*) *yoldii*, *N.* (*Theliostyla*) *albicilla*, *N.* (*T.*) *fulgurans*, *N.* (*T.*) *tessellata*, *N.* (*Lisanerita*) *melanotragus*, and *N.* (*Nerita*) *versicolor*. Outgroup comparison was made with four other neritids: *Clithon retropictum*, *Neripteron violaceum*, *Vitta usnea*, and *Theodoxus fluviatilis*. DNA sequences of each gene were separately aligned using Translator X (for PCGs; Abascal et al. 2010) or MAFFT v7 (rDNA; Katoh and Standley 2013) with default parameters. Removal of alignment-ambiguous sites (identified by Gblocks v.0.91b (Castresana 2000) with all options for a less-stringent selection) resulted in a final dataset of 11 species and 13,394 characters.

The resulting tree (Figure 1) recovered the genus *Nerita* (and its own subfamily Neritinae; Holthuis 1995; Fukumori and Kano 2014) as a robust clade with a maximum bootstrap value (100%). The subgenus *Heminerita* (comprising *N. japonica* and *N. yoldii* alone; Frey 2010) was monophyletic (100%) and sister to a moderately supported clade of the subgenera *Theliostyla* and *Nerita* (84%); the subgenus *Theliostyla* was rendered paraphyletic to *Nerita s.s.*, albeit with a marginal bootstrap value (73%). The mitogenome seems to provide substantial signal for the phylogenetic reconstruction of the Neritidae at the generic and subgeneric levels.

**Figure 1.**
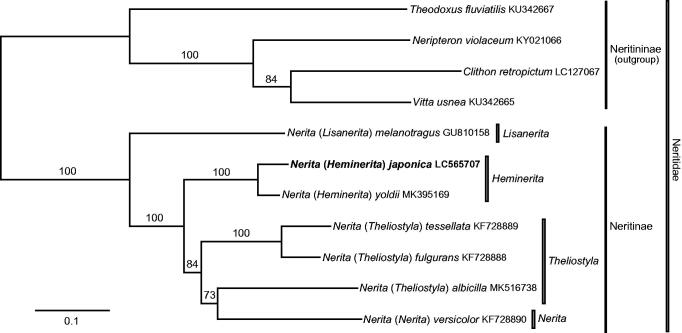
Maximum likelihood phylogeny of genus *Nerita* inferred from nucleotide sequences of 13 protein-cording and 2 ribosomal-RNA genes of mitochondrial genome. Tree reconstruction was performed under GTR + G model in RAxML v.7.4.2 (Stamatakis 2006) with a bootstrap analysis of 1,000 pseudoreplicates. Mitogenome of *Nerita* (*Heminerita*) *japonica* was newly determined; numbers on branches denote bootstrap values in %.

## Data Availability

The data that support the findings of this study are openly available in the DNA Data Bank of Japan (DDBJ) at http://getentry.ddbj.nig.ac.jp/top-e.html, reference number LC565707.
